# Sebaceous neoplasms in Lynch syndrome

**DOI:** 10.1186/1897-4287-9-S1-P7

**Published:** 2011-03-10

**Authors:** Monica Dandapani, Anu Chittenden, Aaron J Stunkel, Margery Rosenblatt, Sapna Syngal, Elena M Stoffel

**Affiliations:** 1 Dana-Farber Cancer Institute, 44 Binney Street, Boston, MA 02115, USA

## Background

Sebaceous neoplasms of the skin (SN) are described in the Muir Torre variant of Lynch syndrome (LS). Guidelines recommend evaluating individuals diagnosed with sebaceous adenomas or sebaceous carcinomas for LS with immunohistochemistry (IHC) for mismatch repair (MMR) proteins and/or microsatellite instability analysis (MSI). The assumption has been that SNs with defective MMR are related to LS.

## Purpose

To describe outcomes of genetic testing for LS among individuals with SN.

## Results

24 individuals with a personal history of SN underwent a genetic evaluation for Lynch syndrome (LS) at Dana-Farber Cancer Institute. 10 had family histories which met Amsterdam criteria, 8 had a personal history of another LS-associated malignancy, 23 had family history of one or more LS-associated cancers, and 1 had no other personal or family history of cancer.

11/24 (46%) probands had pathogenic MMR gene mutations (2 *MLH1* and 9 *MSH2*) and each of these either met Amsterdam criteria or had a personal history of another LS-related cancer.

Of 13 probands without identifiable MMR mutations, 6 had SNs with normal IHC and 7 had abnormal IHC (3 absent MSH2 and MSH6, 2 absent MLH1 and PMS2, 1 absent MLH1 only and 1 absent MSH2 only). One of the probands whose SN showed absence of MSH2 and MSH6 had a family history which met Amsterdam criteria (PREMM score=33%) and the rest had PREMM model scores <5%.

## Conclusions

Although prior reports suggest that MSI/IHC can be useful in screening patients with SNs for LS, we found many of these tumors demonstrate features of abnormal MMR even when family history is not suggestive of LS and genetic testing did not reveal MMR mutation. Further study is needed to determine whether other somatic mechanisms may produce the MMR deficient phenotypes seen in many SNs.

